# Perinatal factors influencing the neonatal hearing screening results

**DOI:** 10.1186/s12887-020-02476-0

**Published:** 2021-01-06

**Authors:** Mahbod Kaveh, Seyedeh Nastaran Mirjalali, Mamak Shariat, Mohammad Reza Zarkesh

**Affiliations:** 1 Department of Pediatrics, Bahrami Hospital, Tehran University of Medical Sciences, Tehran, Iran; 2 Department of Pediatrics, Faculty of Medicine, Tehran University of Medical Sciences, Tehran, Iran; 3 Maternal & Child Health Specialist, Maternal, Fetal and Neonatal Research Center, Tehran University of Medical Sciences, Tehran, Iran; 4 Maternal, Fetal and Neonatal Research Center, Tehran University of Medical Sciences, Tehran, Iran; 5 Department of Neonatology, Yas Women Hospital, Tehran University of Medical Science, Sarv Ave., North Nejatolahi Street, Tehran, 1598718311 Iran

**Keywords:** Transient evoked otoacoustic emissions, Auditory brainstem response, False positive, Neonate

## Abstract

**Background:**

Previous studies have indicated that the majority of cases with “failed” results related to transient evoked otoacoustic emissions (OAE) test have the normal hearing. The present study aimed to assess the possible relationships between perinatal factors and the false-positive OAE results.

**Methods:**

A case-control study was carried out in an Iranian Hospital in 2020. Based on the OAE results on the first day of life, newborns were divided into 2 groups; Control group included subjects with “Pass” OAE results. Every neonate with “Fail” OAE result was referred for auditory brainstem response (ABR). Neonates with bilateral fail OAE but normal ABR results (false-positive OAE) were considered as the case group. All recorded data were analyzed to assess the possible correlations between maternal/neonatal factors and the false-positive OAE results.

**Results:**

One hundred and eighty-one neonates entered the study. Of all included neonates, 87 (48.1%) cases showed bilateral fail OAE and 94 (51.9%) subjects passed the OAE test. Normal ABR results (false-positive OAE) were observed in all cases with bilateral fail OAE. Comparisons of variables affecting the OAE results showed that of all perinatal factors, neonate’s sex (*p* = 0.046) and cesarean section (*p* = 0.003) were the only influencing factors that increased the risk of false-positive OAE results.

**Conclusion:**

Based on the results, the cesarean section delivery and neonate’s male sex increased the risk of false-positive results related to OAE test. Implementing other screening tests such as ABR or Automated ABR as the initial screening test could be suggested for such cases.

## Background

Hearing loss is one of the most frequent congenital anomalies in the neonates. It has been reported that one in every 1000 newborns suffers from congenital bilateral hearing loss [1, 2]. Infants with undetected hearing loss often face significant complications related to emotional, intellectual, linguistic, speech, and social delays [3, 4].

To decrease the impact of adverse effects associated with congenital hearing loss on the infant’s life, early diagnosis and preventive interventions have been recommended [4]. There are different screening methods to evaluate hearing status, as soon as possible, in newborns. Recently transient evoked otoacoustic emissions (TEOAE) and auditory brain stem response (ABR) with high sensitivity and specificity are widely used as universal newborn hearing screening (UNHS) tests. However false-positive results may cause some misinterpretations and parental anxiety [4, 5]. Previous studies indicated that the majority of cases with “failed” results related to screening tests have the normal hearing sense. A high rate of false-positive result ranged from 2.5 to 8% was reported regarding to use of universal newborn hearing screening tests including OAE. The results of the studies have also demonstrated that some factors including ear canal debris, ambient sound, myogenic interference, infant’s position, or activity may falsely influence the results of the screening tests [5, 6]. An investigation demonstrated that of 1000 screened newborns using TEOAE, 18 subjects had impaired two-step OAE results (OAE implementing on first and 10 to 15 days after neonate’s birth). ABR results showed that of these subjects, 12 (1.2%) responses had been false positive [4].

Several studies have evaluated the possible associations between maternal/neonatal factors and false-positive neonatal hearing screening tests; Schwarz et al. showed that 70 newborns who failed OAE, could pass the subsequent ABR. they highlighted that of different maternal/neonatal factors including mother’s health status, age, gravida, parity, amniotic fluid index (AFI), mode of delivery, tobacco & smoking, illegal drug use, alcohol consumption, gestational age, neonate’s gender, head circumference, birth weight, height, and Apgar scores, only smoking and drug use could falsely affect the results [7]. The other study demonstrated that regardless of the mode of delivery, false-positive rates related to the first (81.9%) and second (14.5%) OAE screening test were high among neonates [8]. Kepekci et al. have demonstrated a higher false-positive rate in the first OAE among newborns born by cesarean section (C/S) compared to those born by normal vaginal delivery [9].

False-positive results of hearing screening tests may interfere and postpone the accurate census associated with neonatal hearing loss or impairment. Moreover, requiring repeated screening tests may impose heavy costs on the health system and the family economy. Finally, a false-positive hearing screen may impact some negative psychological and emotional stress on neonates’ parents. Therefore, the present study was carried out to assess the possible relationships between several perinatal factors and false-positive results of the OAE test**.** It is supposed that by identifying such involving factors, implementing of another proper hearing screening test as the initial screening test may eliminate these parents’ discomforts and mentioned complications.

## Methods

### Study design

A case-control study was carried out in Yas Women Hospital affiliated to Tehran University of Medical Sciences (Tehran-Iran) between January and June 2020. The population study was single birth neonates with gestational age > 34 weeks who attended for hearing screening test by OAE (Maico Diagnostics Universal Neonatal Hearing Screening; Germany) on the first day of life. Based on the OAE results, newborns were divided into 2 groups. The control group included subjects with “Pass” results when there was OAE response to the stimulus at > 35 dB Hearing Level (dBHL) [10]. Every neonate with Fail result (no response to the stimulus) was referred for auditory brainstem response (using ABR; The Interacoustics Eclipse; USA) the next day. Neonates with bilateral Fail OAE but normal ABR results (false-positive OAE) were considered as the case group. Both automated OAE and ABR tests were done by an expert audiologist. The case and control groups were matched regarding to age and sex. Newborns with failed both OAE and ABR tests as well as having the risk factors associated with hearing loss (Maternal infections, Hyperbilirubinemia, congenital head and neck abnormalities or syndromes) were also excluded from the study.

Demographic data related to all neonates and their mothers including maternal age, level of education, parity, gravid, type of delivery, prenatal corticosteroid administration, prenatal complications and underlying diseases, amniotic fluid index (AFI), gestational age, the first and fifth minutes Agar scores, anthropometric measures, and history of hospitalization were extracted from medical records and recorded.

All recorded data related to the results of hearing screening tests as well as maternal/ neonatal data were analyzed to assess the possible correlations between some maternal-neonatal factors and false positive OAE result.

### Ethical considerations

Ethics approval was obtained from the institutional review board of Tehran University of Medical Sciences according to Helsinki declaration (IR.TUMS.VCR.REC.1398.061). All participants’ parents gave written consent before enrollment. Participants’ data were considered confidential and no extra cost was imposed on our participants.

### Sample size

Schwarz et al. [7] in a study (determining the affecting factors on false positive OAE test) showed 10% of false positive OAE in newborns were associated with maternal variables like drug usage, while this rate among the control group was 1.3%. Based on these parameters, using formula for estimation of a proportion between two independent groups with a power of 80% and an alpha error of 0.05, 94 subjects in each group (for achieving the effective factor on false positive OAE) were included.

$$ n=2\frac{{\left({z}_{1-\frac{\alpha }{2}}+{z}_{1-\beta}\right)}^2\overline{p}\overline{q}}{\left({p}_1-{p}_2\right)} $$

$$ \upalpha =0.05,\upbeta =0.2,{\mathrm{P}}_1=0.10,{\mathrm{P}}_2=0.013,\mathrm{Z}1\hbox{-} \mathrm{a}/2=1.96,\mathrm{Z}1\hbox{-} \mathrm{B}=0.84,\mathrm{p}=0.055,\mathrm{n}=94 $$

### Data analysis

Analyses were statistically performed by using software package SPSS Version 18. Quantitative and qualitative variables were reported by mean ± SD and percent, respectively. Chi-square, Non-parametric Kolmogorov-Smirnov test (determining normal or non-normal distribution of variables), and Mann-Whitney test (for comparison of quantitative variables with non-normal distribution) were used for determining associations between different maternal/ neonatal factors and false positive OAE test. Binary logistic regression test was also used to eliminate the effects of confounding factors. The level of significance was considered as *P* < 0.05.

## Results

One hundred and eighty-one neonates entered the study. None of mothers had smoking, alcohol or drug consumption. The mean of mothers’ age, gravid, parity and amniotic fluid index were 30.1508 ± 5.24401 years, 1.966 ± 0.8975, 1.740 ± 0.7152 and 13.271 ± 3.252 (Min: 1.195, Max: 25) Cm. The Minimum, Maximum and the mean of neonates’ gestational age were 34, 40^+ 6^ and 38.423 ± 1.107 weeks. The mean of neonate’s birth weight, Height and head circumference were 3381.655 ± 2305.851 (min; 2270.00 & max; 33,680.00) grams, 50.792 ± 2.1956 and 34.653 ± 1.1633 Cm, respectively. Detailed demographical and obstetrical data are shown in Table 1.

**Table 1:** All participants’ demographical and obstetrical data

Variables	Number	Percent
**Level of educations**		
Lower diploma	25	13.9
Diploma	94	52.2
Higher diploma	61	33.8
Maternal health status		
Healthy	118	65.2
None healthy	63	34.8
**Underlying disease in none healthy group**		
High blood pressure	4	2.2
Urinary tract Infection	1	0.6
Diabetes	18	9.9
Thyroid	33	18.2
other	7	3.9
**Corticosteroid administration**		
Yes	20	11.0
No	161	89.0
**Occupation**		
Employed	13	7.2
Unemployed	168	92.8
**Mode of delivery**		
Cesarean section	106	59.6
Vaginal delivery	72	40.4
**Neonate’s sex**		
Male	99	56.9
Female	75	43.1
**Amniotic fluid status**		
Oligohydramnios	1	0.6
Polyhydramnios	1	0.6
Normal	175	98.9
**Apgar scores**		
First minute	8.9945 ± .07433	
Fifth minute	9.9945 ± .07433	
**OAE**		
Left Pass	93	51.7
Right Pass	93	51.7
Both fail	87	48.1
Both pass	94	51.9
Left fail	87	48.3
Right fail	87	48.3

### Comparison of qualitative and quantitative data between the case and control groups

Seven cases with Fail OAE results did not attend for ABR. Finally, of all 181 included neonates, 87 (48.1%) cases showed bilateral Fail OAE and 94 (51.9%) subjects as the control group passed the OAE test. All 87 cases with bilateral Fail OAE and normal ABR results (false-positive OAE) were included. As the results are demonstrated in Tables 2 & 3, false-positive OAE results were more common in male cases compared to the females (*p* = 0.009; OR = 2.278, 95% CI: 1.232, 4.212). There was also a significant difference between groups regarding to type of delivery; more neonates in the case group were born by cesarean section (*p* = 0.002; OR = 2.649, 95% CI: 1.425, 4.925). No significant differences were observed between groups regarding to maternal occupation (*p* = 0.776), educations (*p* = 0.956), underlying disease (*p* = 0.116), corticosteroid administration (*p* = 0.101)). There were also no significant differences between groups with respect to neonatal gestational age (*p* = 0.074), 1st (*p* = 0.299**)** & 5th (*p* = 0.299**)** minutes Apgar scores, birth weight (*p* = 0.233), height (*p* = 0.280) and head circumference (*p* = 0.136).

**Table 2:** Comparison of qualitative variables between the case and control groups

	Case ***n*** = 87	Control ***n*** = 94	***P*** value*
**Level of educations**			
Lower diploma	12 (14)	13 (13.8)	0.956
Diploma	44 (51.2)	50 (53.2)	
Higher diploma	30 (34.9)	31 (33)	
**None healthy group with underlying disease**			
High blood pressure	4 (4.6)	0 (0)	0.116
Urinary tract	1 (1.1)	0 (0)	
Diabetes	6 (6.9)	12 (12.8)	
Thyroid	14 (16.1)	19 (20.2)	
other	2 (2.3)	5 (5.3)	
**Healthy group**	60 (69.0)	58 (61.7)	
**Corticosteroid administration**			
Yes	6 (6.9)	14 (14.9)	0.101
No	81 (93.1)	80 (85.1)	
**Occupation**			
Employed	7 (8.0)	6 (6.4)	0.776
Unemployed	80 (92.0)	88 (93.6)	
**Type of delivery**			
Cesarean section	62 (71.3)	44 (48.4)	0.002
Vaginal delivery	25 (28.7)	47 (51.6)	
**Neonate’s sex**			
Male	57 (67.1)	42 (47.2)	0.009
Female	28 (32.9)	47 (52.8)	
**Maternal health status**			
Healthy	60 (69.0)	58 (61.)	0.305
None healthy	27 (31.0)	36 (38.3)	
**Amniotic fluid status**			
Oligohydramnios	1 (1.2)	0 (0.0)	0.326
Polyhydramnios	1 (1.2)	0 (0.0)	
Normal	82 (97.6)	93 (100)	

*Chi-square test

**Table 3:** Comparison of quantitative variables between the case and control groups

	Case group Mean ± std	Control group Mean ± std	***P*** value*
Mother**’s** age (Years)	30.183 **±** 5.386	30.119 **±** 5.135	0.918
Amniotic index (Cm)	12.935 **±** 3.658	13.109 **±** 2.853	0.337
Gravid	1.965 **±** 0.881	1.966 **±** 0.917	0.916
Parity	1.781 **±** 0.722	1.700 **± 0**.710	0.405
Gestational age (Weeks)	38.289 **±** 1.062	38.538 **±** 1.137	0.074
1st minute Apgar score	8.988 **±** 0.107	9.000 **±** 0.000	0.299
5th minute Apgar score	9.9885 **±** 0.107	10.000 ± 0.000	0.299
Birth weight (g)	3262.1512 ± 435.89026	3490.989 ± 3167.768	0.233
Height (Cm)	50.6221 ± 2.19080	50.951 ± 2.200	0.280
Head (Cm)	34.8140 ± 34.8140	34.500 ± 1.121	0.136

*Mann-Whitney test

Adjusting confounding factors, comparison of variables affecting OAE results were also done by logistic regression test (Table 4). The results showed that of all perinatal factors, neonate’s sex (*p* = 0.046) and cesarean section (*p* = 0.003) were the only influencing factors for false-positive OAE results.

**Table 4:** Comparison of maternal/neonatal variables affecting false-positive OAE results (Binary logistic regression)

Source	Independent Variable	B	*P* value	CI_95%_
Neonatal factors	- Gestational age	0.206	0.203	0.895–1.686
	- 1st minute Apgar	20.300	1.000	0.000
	- Sex	0.681	0.046	1.011–3.862
	- Weight	0.000	0.611	−0.002-0.000
	- Height	0.082	0.325	0.922–1.279
	- Head	− 0.295	0.056	0.550–1.007
Maternal factors	- Amniotic index	−0.001	0.870	0.989–1.009
	- Gravid	0.191	0. 566	0.630–2.329
	- Parity	−0.441	0.310	0.274–1.508
	- Occupation	0.025	0.972	0.246–4.270
	- Type of Delivery	1.042	0.003	1.409–5.709
	- Age	0.051	0.169	0.978–1.133
	- Amniotic fluid index	−0.001	0.870	0.989–1.009
	- Education	−0.144	0.349	0.641–1.170

## Discussion

In the present study, possible relationships between several perinatal factors and false-positive results related to the OAE test were assessed. By identifying such influencing factors, initially using a more advanced screening test like ABR can eliminate the defect associated with OAE screening test.

Our results showed that of all perinatal factors, neonate’s sex was a significant influencing factor on the false-positive OAE test; the majority of the cases were male (67.1% vs. 32.9%). We suppose that this sex-related discrepancy may arise from differences in male and female’s skull bones as well as skull structures. A former study has reported that the amplitudes of the otoacoustic emissions for female neonates are greater and more perceptible than their male counterparts [11]. Other studies have also shown that the failed results related to NHS tests were more frequent in male subjects; Lima et al. have reported that 12% of participants in their study failed the NHS test; of them 55.27% were male [12]. Other investigation showed that of 88 screened neonates, 39.77% failed the NHS while the numbers of male subject were significantly higher than females (62.86% vs. 51.43%) [13]. In accordance with our finding da-Silva Reis et al. demonstrated that the fail results associated with the first hearing screening test were significantly frequent among male newborns compared to the females (*n* = 34; male: 28.3% vs. female: 11.7%, *p* = 0.0128). Of all these 34 cases, more male neonates passed the rescreening ABR test than the females (17.6% vs. 14.7%) [14].

The results in the current study have also indicated that there was a significant difference between case and control groups regarding to type of delivery; more neonates with false-positive OAE results had been born by cesarean section. The reason may correlate to accumulation of wax or amniotic fluid in the external and middle ear in the cesarean cases. Unlike the OAE test, the ABR test is not affected by such factors [4, 15]. In addition, it is supposed that in the cesarean cases, anesthesia and the time of screening after that may affect the OAE results [16]. According to such findings, we suggest the implementing of ABR test as the first screening test for neonates born by cesarean section. By using this test, rescreenining might be avoided. Compatible to our findings, Xiao et al. have indicated that cesarean section and early screening within the first 42 h after that have significantly increased the false-positive OAE results among neonates [17]. Smolkin et al. demonstrated that cesarean section could increase the risk of failure of OAE test by 3.2 times compared to vaginal delivery. They have proposed that to decrease the false-positive failure rates among neonates born by cesarean section, OAE screening test should be performed beyond 48 h after the birth [15]. Oghan et al. reported that of 6044 newborns born by cesarean section, 424 neonates failed the OAE test while 6.1% of them could pass the ABR test. Of 4723 subjects with vaginal delivery, 227 neonates failed the OAE test, of them 4.3% could pass the ABR test. The authors indicated that although false-positive results in the cesarean group were higher than the normal vaginal delivery group; the difference between two groups was not significant [18]. Güven also did not find any significant association between false-positive hearing screening result and the mode of delivery, however the author showed that after fluid resorption in the ear, success rate related to the first OAE was significantly increased [8]. Tabrizi et al. have also noted that for higher success rate, the first OAE test after cesarean delivery should be postponed [19]. In contrast to our finding, Farahani et al. have demonstrate that false-positive OAE results in the vaginal delivery group was significantly higher than the cesarean group (15.5% vs. 9.5%) [20].

According to the results, we could not find any significant relationships between false-positive OAE results and other perinatal factors. However further studies with larger sample size, implementing of different screening methods in variable postnatal times may provide more beneficial data.

## Conclusion

In summary based on the results, the cesarean section delivery and neonate’s male sex increased the risk of false-positive results related to OAE test. Implementing other screening tests such as ABR or Automated ABR as the initial screening test could be suggested for such cases. Using these methods as the first screening tool may be cost effective by avoiding of rescreenining and also reduces parents’ anxiety related to false-positive results. Moreover, with regard to high rates of false-positive rates in the implementing of OAE test, the performing the second screening modality for the failed OAE cases should be considered.

## Data Availability

The datasets related our study is available from the corresponding author on reasonable request.

## Abbreviations

UNHS: Universal newborn hearing screening
OAE: Otoacoustic emissions
TEOAE: Transient evoked otoacoustic emissions
ABR: Auditory brainstem response
AFI: Amniotic fluid index
C/S: Cesarean section
NVD: Normal vaginal delivery
Dbhl: Decibels Hearing Level

## References

[1] Valse D, Nagarathna HK (2017). Efficacy of TEOAEs and BERA as screening tools for deafness in newborn. Int J Otorhinolaryngol Head Neck Surg.

[2] Qi B, Cheng X, En H, Liu B, Peng S, Zhen Y, Cai Z, Huang L, Zhang L, Han D (2013). Assessment of the feasibility and coverage of a modified universal hearing screening protocol for use with newborn babies of migrant workers in Beijing. BMC Pediatr.

[3] Crouch E, Probst J, Bennett K (2017). Evaluating loss to follow-up in newborn hearing Screeningin a southern state. JEHDI.

[4] Yousefi J, Ajalloueyan M, S Amirsalari S, Hassanali Fard M (2013). The specificity and sensitivity of transient Otoacustic emission in neonatal hearing screening compared with diagnostic test of auditory brain stem response in Tehran hospitals. Iran J Pediatr.

[5] Poulakis Z, Barker M, Wake M (2003). Six month impact of false positives in an Australian infant hearing screening programme. Arch Dis Child.

[6] Clemens CJ, Sherri A (2001). Davis. Minimizing false-positives in universal newborn hearing screening: a simple solution. Pediatrics.

[7] Schwarz Y, Kaufman GN, Daniel SG (2017). Newborn hearing screening failure and maternal factors during pregnancy. Int J Pediatr Otorhinolaryngol.

[8] Gülseven GS (2019). The effect of mode of delivery on newborn hearing screening results. Turk Arch Otorhinolaryngol.

[9] Hamdi Kepekci A, Bestemi KA (2019). Effect of delivery modes on hearing screening results. Glob J Otolaryngol.

[10] Amini E, Farahani Z, Rafiee Samani M, Hamedi H, Zamani A, Karimi Yazdi A, Nayeri F, Nili F, Rezaeizadeh G (2014). Assessment of hearing loss by OAE in asphyxiated newborns. Iran Red Cres Med J.

[11] Durante AS, Carvallo RM, da Costa FS, Soares JC (2005). Characteristics of transient evoked otoacoustic emissions in newborn hearing screening program. Pro Fono.

[12] Lima MCMP, Rossi TRF, Françozo MFC, Marba ST, Lima GML, Santos MFC (2010). Detection of hearing loss in neonates of a public hospital. Rev Soc Bras Fonoaudiol.

[13] Maia RM, Silva MAM, Tavares PMB (2012). Newborn hearing health: speech therapy acting on family health strategy. Rev CEFAC.

[14] da Silva RF, de Oliveira GC, De Conto J, Iantas M, Lüders D, Marques J (2019). Hearing assessment of neonates at risk for hearing loss at a hearing health high complexity service: an electrophysiological assessment. Int Arch Otorhinolaryngol.

[15] Smolkin T, Mick O, Dabbah M, Blazer S, Grakovsky G, Gabay N (2012). Birth by cesarean delivery and failure on first otoacoustic emissions hearing test. Pediatrics.

[16] Khoza-Shangase K, Joubert K (2011). The influence of epidural anesthesia on new-born hearing screening: a pilot study. J Pharm Bioall Sci.

[17] Xiao T, Li Y, Xiao L, Jiang L, Hu Q (2015). Association between mode of delivery and failure of neonatal acoustic emission test: a retrospective analysis. Int J Pediatr Otorhinolaryngol.

[18] Oghan F, Guvey A, Fatih Topuz M, Erdogan O, Guvey H (2020). Effects of vaginal birth versus caesarean section on hearing screening results in a large series from the Aegean region. Int J Audiol.

[19] Tabrizi A, Asadi M, Barati B, Shahabi M (2017). Birth by cesarean delivery on newborn screening test: A RETROSPECTIVE STUDY. IJLPR.

[20] Farahani F, Hamidi Nahrani M, Seifrabiei MA, Emadi M (2017). The effect of mode of delivery and hospital type on newborn hearing screening results using Otoacoustic emissions: based on screening age. Indian J Otolaryngol Head Neck Surg.

